# Identification of exosomes and its signature miRNAs of male and female ***Cynoglossus semilaevis***

**DOI:** 10.1038/s41598-017-00884-4

**Published:** 2017-04-13

**Authors:** Zhanpeng Sun, Tong Hao, Jinze Tian

**Affiliations:** 1 College of Life Sciences, Zhejiang University, Zhejiang, 310058 P.R. China; 2 Tianjin Key Laboratory of Animal and Plant Resistance/College of Life Sciences, Tianjin Normal University, Tianjin, 300387 P.R. China

## Abstract

Exosomes are small membrane particles which are widely found in various cell lines and physiological fluids in mammalian. MicroRNAs (miRNAs) enclosed in exosomes have been identified as proper signatures for many diseases and response to therapies. However, the composition of exosomes and enclosed miRNAs in fishes has not been investigated. *Cynoglossus semilaevis* is an important commercial flatfish with ambiguous distinction between males and females before sex maturation, which leads to screening difficulty in reproduction and cultivation. An effective detection method was required for sex differentiation of *C. semilaevis*. In this work, we successfully identified exosomes in *C. semilaevis* serum. The analysis of nucleotide composition showed that miRNA dominated in exosomes. Thereafter the miRNA profiles in exosomes from males and females were sequenced and compared to identify the signature miRNAs corresponding to sex differentiation. The functions of signature miRNAs were analyzed by target matching and annotation. Furthermore, 7 miRNAs with high expression in males were selected from signature miRNAs as the markers for sex identification with their expression profiles verified by real time quantitative PCR. Exosomes were first found in fish serum in this work. Investigation of marker miRNAs supplies an effective index for the filtration of male and female *C. semilaevis* in cultivation.

## Introduction

Exosomes are small membrane particles originated from the multivesicular bodies with the function that conveying biological materials to surrounding cells^1^. Exosomes were firstly found in the human neoplastic cell lines^2^, and then the cultured rat glioma C6 cells were found to secrete exosomes into the culture medium^3^. Currently, exosomes have been found in many physiological fluids in mammalian such as urine^4^, plasma^5^, cerebral fluid^6^ and even in organs such as thymus^7^. Moreover, exosomes are found to be secreted from various cells including B cells^8^, T cells^9^, dendritic cells^10^, platelets^11^, the Schwann cells^12^, tumor cells^13^, cardiomyocytes^14^, endothelial cells^15^ and stem cells^16^. Most exosomes contain a set of proteins such as heat shock proteins, HSP70^17^ and HSP90^18^, certain members of the tetraspanin superfamily of proteins, especially CD9, CD63, CD81 and CD82^19^, which enable the identification of exosomes. Although the exact functions of exsomes are still unclear, exosomes have been proved to be involved in many different biological processes, such as follicular maturation^20^, RNA and protein transport^21^, biomarkers of pathologies^22^ and also potential targets of therapy in different neoplastic and degenerative diseases^23^.

Exosomes are enriched with various nucleic acids including mRNAs, microRNAs (miRNAs) and other noncoding RNAs^24^. MiRNAs are endogenous, noncoding RNA molecules of about 22 nucleotides in length that regulate gene expression in wide range of biological process such as normal development, cell growth, differentiation and apoptosis^22^. They regulate posttranscriptional gene expression through targeting mRNAs by directing cleavage or repressing translation^25^. As exosomes have been proved to naturally carry RNA between cells^26, 27^, it is speculated that the exchange of genetic information among tumor cells is achieved by the transfer of exosome-shuttle miRNAs^28^. MiRNAs carried by exosomes were released by a donor tumor cell into extracellular environment and then transferred into recipient cells^4^. The transfer of exosome-shuttle miRNAs plays a role of communication and posttranscriptional regulation between cells^29^. Furthermore, miRNAs have been identified to be extremely stable and readily extracted from various types of cell lines or tissues, which makes them the proper signatures for many diseases and response to therapies in mammals^30^. Studies about miRNAs have covered many kinds of species and tissues^31–33^. The miRNA/isomiR expression with gender difference were even analyzed in human tumor cells^34^, which provides a novel view for the study of human tumor. However, in fish, only the exosomes from cultured salomon leukocyte were identified^35^, but the composition of nucleotides and transferred proteins in fish exosomes are still unclear.

*Cynoglossus semilaevis*, also named as half-smooth tongue sole, is an important marine commercial fish specie that is widely distributed in Chinese coastal waters. This specie shows no obvious difference between male and female fishes before sex maturation. Although *C. semilaevis* exhibits a sexually dimorphic growth with the females growing much faster than males and finally sizes reaching to 2–4 times of male size^36^, the distinguish of male and female fishes merely on body sizes causes large amount of waste due to the individual growth difference. Therefore, a reliable detection method for sex differentiation of *C. semilaevis* is important to enhance the efficiency of reproduction. Currently, some conceptions have been studied with the intention of distinguishing the males and females, such as ZW sex chromosome evolution and benthic lifestyle adaptation^37^, sexual reversal^38^ and sex determination genes^39, 40^. However, an effective index for sex differentiation is still indeterminate.

In this work, we firstly identified the existence of exosomes in *C. semilaevis* serum. The nucleotide composition of exosomes was then analyzed. Furthermore, miRNA profiles in exosomes from males and females were sequenced and compared to investigate the signature miRNAs for sex differentiation. The functions of these signature miRNAs were investigated by target gene matching. Markers for sex differentiation were further selected from the signatures and verified by real time quantitative PCR, which provide an effective reference index on molecular level for the differentiation of male and female *C. semilaevis*.

## Results

### Identification of exosomes in *C. semilaevis* serum

**Figure 1:**
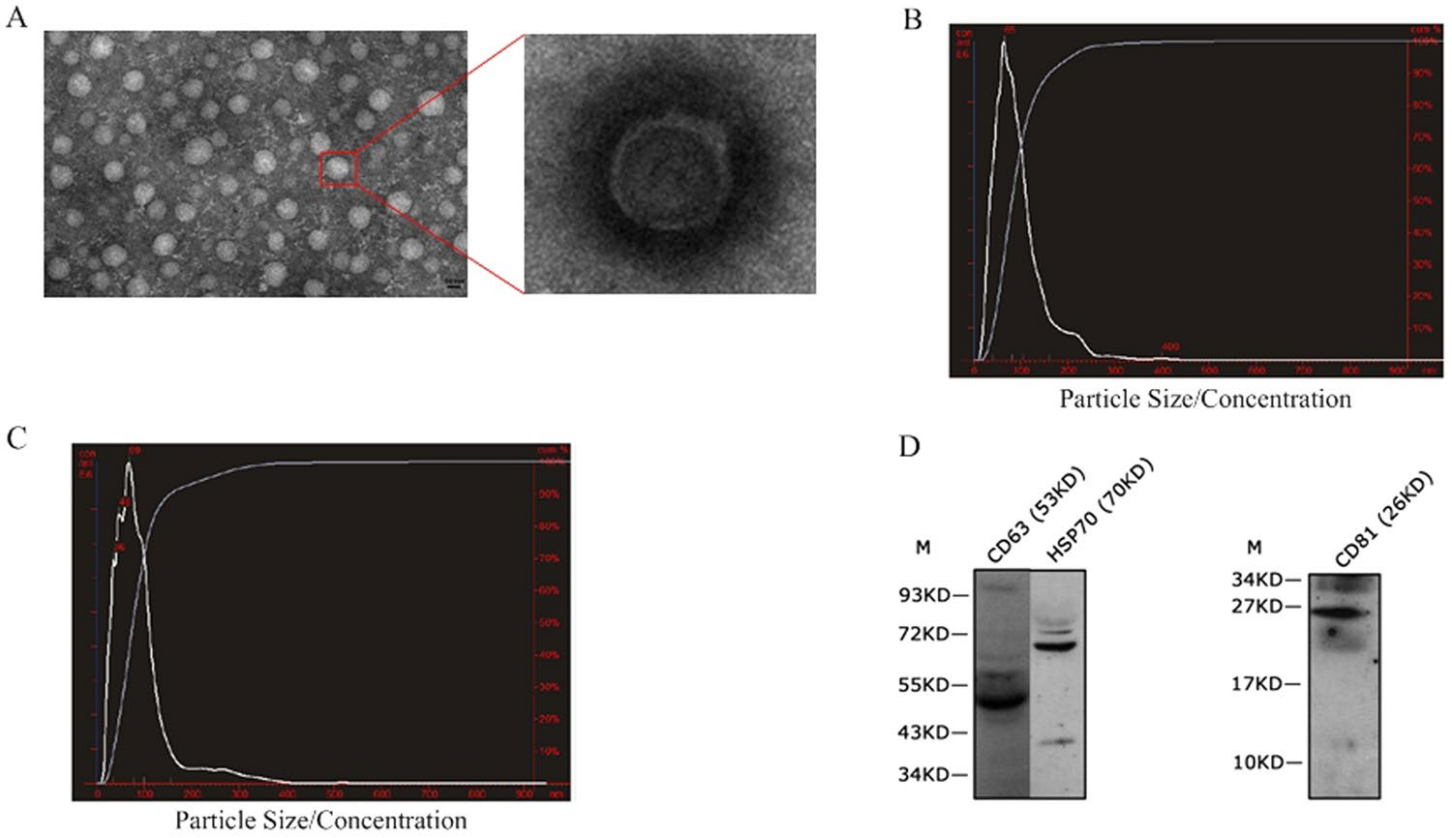
Isolation and identification of exosomes from *C. semilaevis* serum. (**A**) Electron microscope images of exosomes; (**B**) Particle size distributions and concentration of exosomes in males analyzed by NTA; (**C**) Particle size distributions and concentration of exosomes in females analyzed by NTA; (**D**) Identification of CD63, HSP70 and CD81 by Western Blotting.

In order to generate sufficient exosomes for total RNA analysis, we collected about 40 ml serum from 10 tales of male and female fishes, respectively. Exosomes were isolated with differential centrifugation (see method section). Exosomes purified from male and female fishes were observed by transmission electron microscopy to be small (30–120 nm) spherical vesicles (Fig. 1A). In addition, we further analyzed the sizes, size distributions and concentrations of our exosome preparations with Nanopartical Tranking Analysis (NTA), which is a real-time visualization method that can be used to rapidly identify the size, concentration and protein biomarkers of exosomes in biological fluids. The particles with diameters distributing from 30 to 120 nm takes over 80% of all the particles with the mean size as 95 nm and 93 nm in males and females, respectively (Fig. 1B and C), which is consistent with the characteristic size range (30–120 nm) of exosomes^41^. The mean concentration is 3.3 × 10^9^ particles/ml. Based on these measurements, it seems that our exosome preparations isolated from male and female *C. semilaevis* serum contain a heterogeneous mixture of exosome and microvesicles collected with high speed ultracentrifugation protocols^42^, which is consistent with the standpoint that several diverse population of vesicles (including exosomes, microvesicles, ectosomes, membrane particles, exosome-like vesicles, and apoptotic vesicles) are present in many exosome preparations obtained by differential ultracentrifugation^43^.

To confirm the existence of exosomes in our preparations, we investigated the presence of four common exosome markers by Western Blotting, including the tetraspanins CD63, CD81, CD9 and the heat shock protein HSP70^43^. The existence of CD63, CD81 and HSP70 were clearly observed by western blotting analysis system with CD63 in 53KD, HSP70 in 70KD and CD81 in 26KD (Fig. 1D), whereas CD9 had no obvious strip. These observation and analysis of the exosome preparations showed obvious characteristic of exosomes, which proved the existence of exosomes in *C. semilaevis* serum.

### Nucleotide composition of exosomes

**Figure 2:**
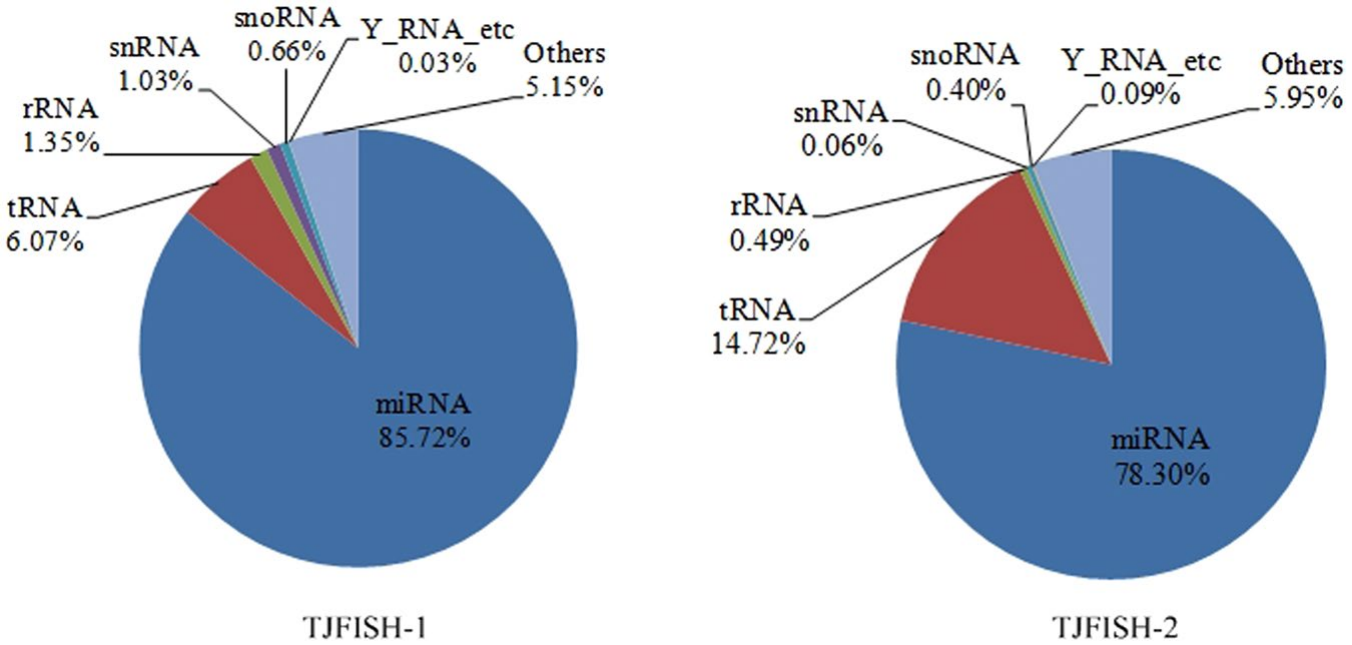
Nucleotide composition of exosomes in TJFISH-1 and TJFISH-2.

Exosomes are identified to be enriched with various nucleic acids including mRNAs, miRNAs and other noncoding RNAs in mammalian^24^. However, the nucleotide composition of exosomes in fish has not been studied. Therefore, we sequenced the total RNA in isolated exosomes from male (TJFISH-1) and female (TJFISH-2) *C. semilaevis*, respectively. The total RNA reads were found quite different in TJFISH-1 and TJFISH-2 with only 22.46% (203452/905922) common reads in all the RNAs from exosomes. The unique RNA reads takes about 66.65% (406553/610005) and 59.26% (295937/499389) of TJFISH-1 and TJFISH-2, respectively. The nucleotide in exosomes is composed of miRNA, tRNA, rRNA, snRNA, snoRNA and Y_RNA, in which miRNA dominates in both TJFISH-1 and TJFISH-2 (Fig. 2). With the comparison to miRBase database^34^, we identified 723 and 662 known miRNAs from TJFISH-1 and TJFISH-2 respectively, in which 635 are common miRNAs (Supplementary File 1). In addition, 20 novel miRNAs and 17 novel pre-miRNAs were predicted in TJFISH-1, whereas 13 novel miRNAs and 12 novel pre-miRNAs were predicted in TJFISH-2 (Supplementary File 2).

### miRNA expression profiles

**Figure 3:**
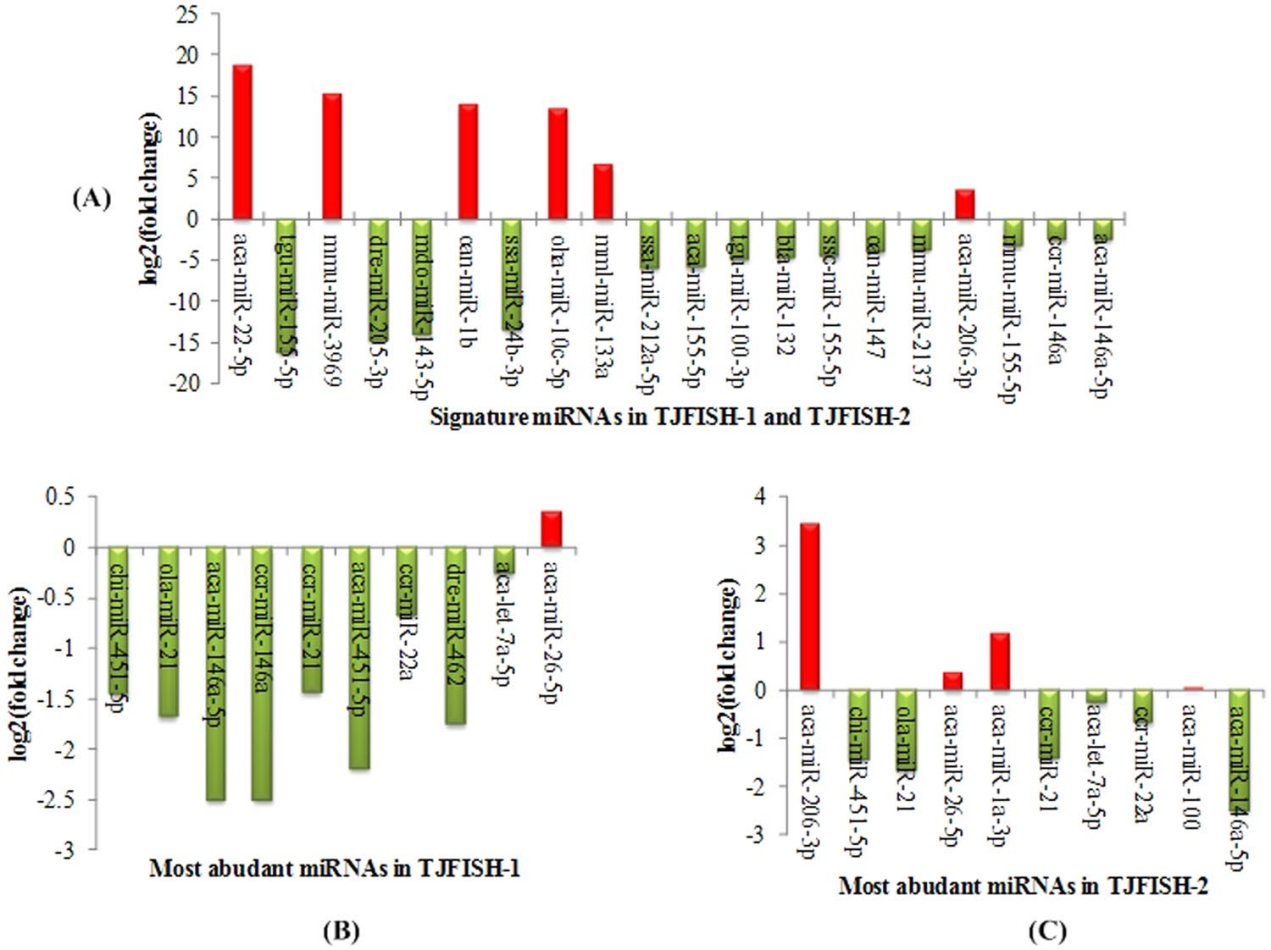
Expression of selected miRNAs in *C. semilaevis*. (**A**) Expression of 20 signature miRNAs in TJFISH-1 and TJFISH-2. (**B**) Expression of 10 most abundant miRNAs in TJFISH-1. (**C**) Expression of 10 most abundant miRNAs in TJFISH-2. Red and green colors indicate up and down regulation of miRNAs in TJFISH-2 compared to TJFISH-1, respectively.

MiRNAs have been found conserved throughout evolution and performing important roles in cell differentiation and development of invertebrates, plants, animals and humans. The stability of miRNAs is possibly because that they are enclosed within exosomes^20^. We compared the miRNA profiles to investigate the difference between male and female *C. semilaevis*. Totally 270 differentially expressed miRNAs were identified according to the criteria in the methods section (Supplementary File 3), in which 23 were from TJFISH-1, 24 were from TJFISH-2 and 223 were from both groups. In these miRNAs, 76 were up regulated and 194 were down regulated in TJFISH-2 compared to TJFISH-1. In all the differentially expressed miRNAs, 20 typical miRNAs were identified as signatures, including (1) 8 miRNAs with expression in one group but no expression in the other; (2) 9 miRNAs with a non-zero expression in one group but a much higher expression in the other; (3) 3 miRNAs with quite high expression (higher than 3000RPM) in both groups but high fold changes. The expression profiles of these miRNAs were shown in Fig. 3A.

### Target prediction for signature miRNAs and function analysis

MiRNAs influence biological process by regulating the expression of multiple target genes when exosomes containing miRNA cargo are released by a donor cell into the extracellular environment and then functionally transferred to recipient cells^44^. For the purpose of investigating the influence of miRNAs on the sex differentiation of *C. semilaevis*, we predicted the target genes of the 20 signature miRNAs and 10 most abundant miRNAs in males and females, respectively (Fig. 3B and C). As there are several common miRNAs in the three groups, totally 31 miRNAs were analyzed.

**Figure 4:**
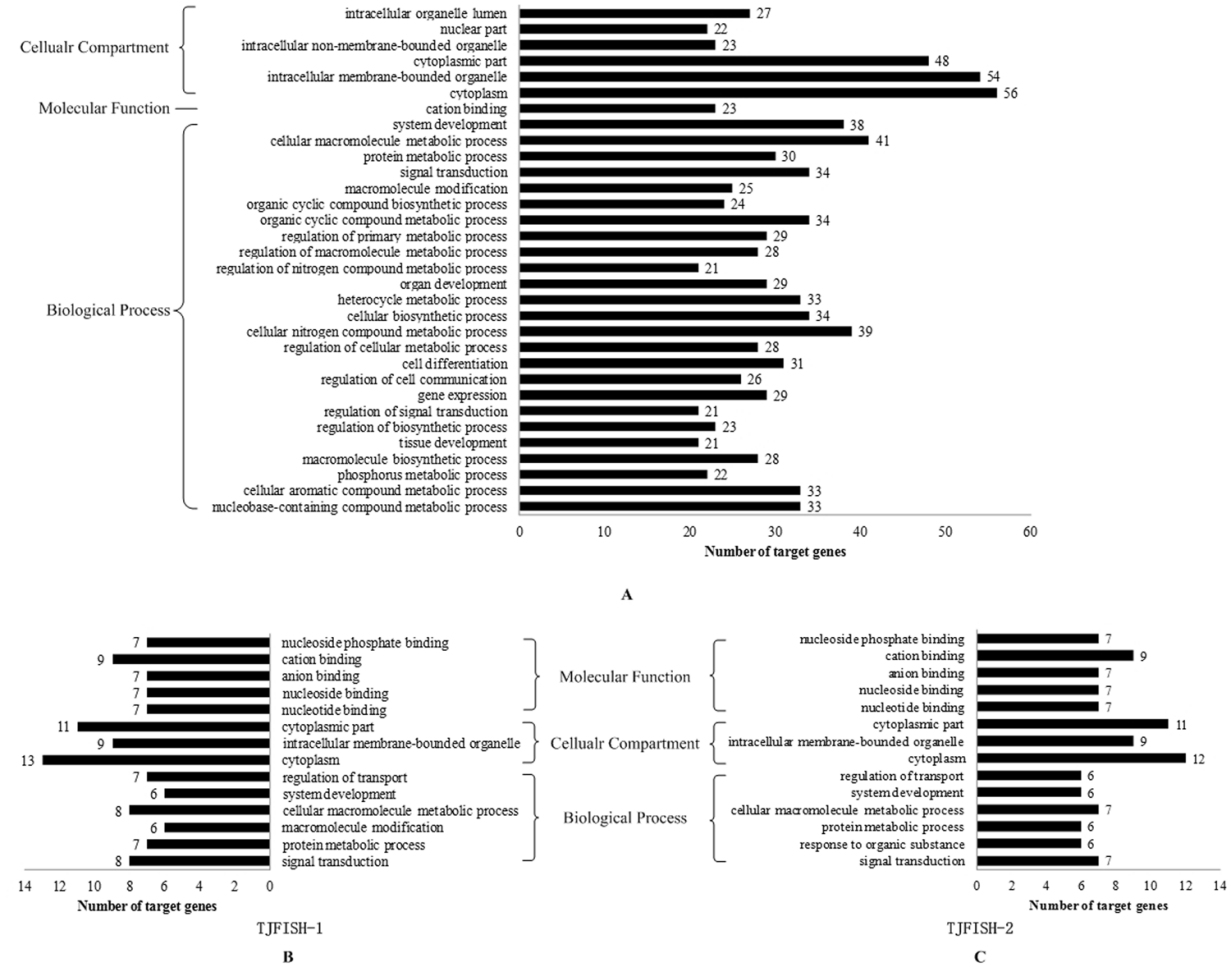
GO analysis of targets genes related to signature miRNAs and abundant miRNAs in TJFISH-1 and TJFISH-2. (**A**) Signature miRNAs; (**B**) Abundant miRNAs in TJFISH-1; (**C**) abundant miRNAs in TJFISH-2.

With the intersection of miRanda, PITA and RNAhybrid software, 93 target genes were found, including 79 for the differentially expressed miRNAs, 16 for males and 15 for females. 17 target genes were overlapped among these three groups. Three miRNAs, aca-miR-155-5p, aca-miR-206-3p and mmu-miR-155-5p, have no common target genes predicted by all three software. The GO annotation of the target genes for differentially expressed miRNAs and top 10 abundant miRNAs in TJFISH-1 and TJFISH-2 were shown in Fig. 4 and Supplementary File 4. According to GO term categories generated from the genes targeted by the signature miRNAs, most target genes predicted to be regulated by exosome miRNAs participate in the cellular macromolecule metabolic process, cellular nitrogen compound metabolic process, system development, signal transduction process, organic cyclic compound metabolic process and cellular biosynthetic process. The target genes mostly locate in cytoplasm, cytoplasmic part and intracellular membrane-bound organelle with the most part of genes function as cation binding protein. The large amount of target genes functions in the biological process have close relationship with sex differentiation, including system development, organ development and cell differentiation, indicating that these related miRNAs has strong possibility to be related to the sex differentiation process in *C. semilaevis*. Besides, 5, 5 and 7 genes were annotated to be male sex differentiation, female sex differentiation and sex differentiation. These genes related to 5 signature miRNAs: dre-miR-205-3p, mml-miR-133a, oha-miR-10c-5p, ssa-miR-212a-5p and aca-miR-100, respectively. The sex differentiation possibly preformed through the signal transduction, macromolecular biosynthesis and regulation, nitrogen and heterocycle metabolism as lots of targets genes distributed in these biological processes. The function annotation for the target genes regulated by the 10 most abundant miRNAs in males and females are mainly the same. The only difference is that miRNAs prefer to function in macromolecule modification in males rather than response to organic substance in females.

**Figure 5:**
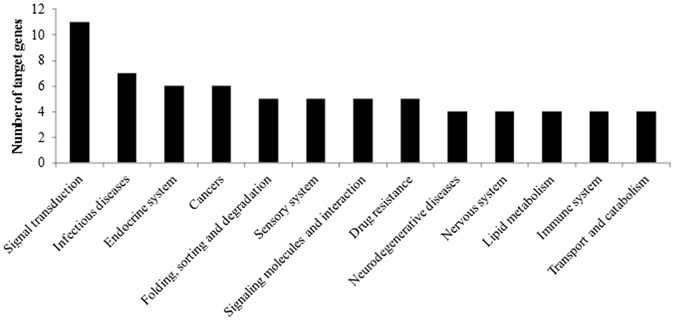
Distribution of target genes in KEGG subsystems.

To further analysis the function of targets genes, we investigated the KEGG pathways of these genes. However, not all the genes can be annotated with a certain KEGG pathway. In all the 93 target genes, 24 were found KEGG pathway annotation. These genes distributed in 98 pathways and 40 subsystems. Figure 5 shows the subsystems with more than three genes. The most enriched subsystem is signal transduction, and besides, signaling molecules and interaction is also included in the enriched subsystems. As only one quarter of the target genes can be annotated with KEGG pathways, the pathway analysis partly reflects the function of the signature miRNAs. The signal-related function is highlighted among all the subsystems, which is consistent with GO function analysis of target genes.

### Real time quantitative PCR

**Figure 6:**
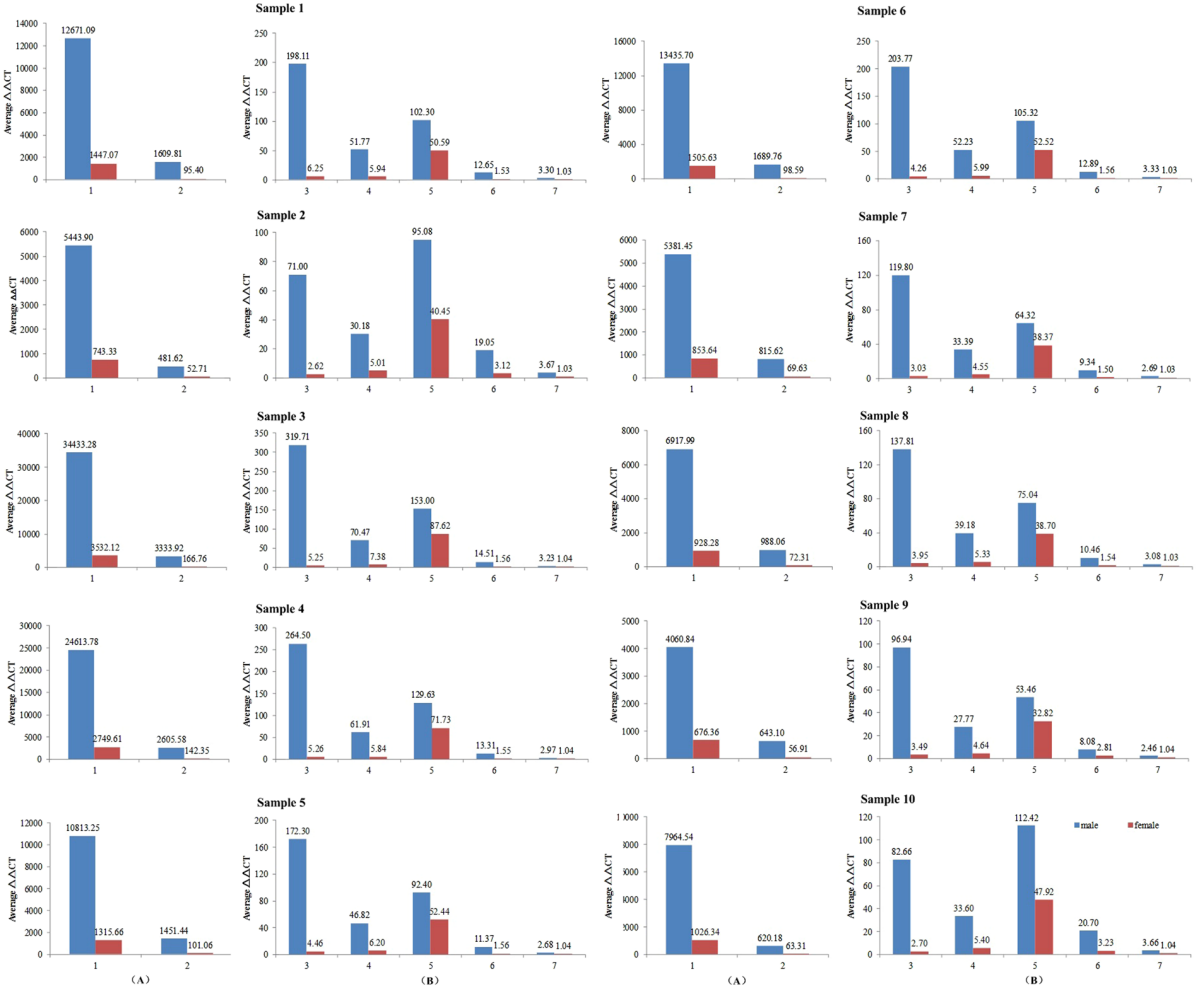
Expression of marker miRNAs measured by RT-qPCR. 1: Aca-miR-155-5p; 2: Bta-miR-132; 3: Mmu-miR-155-5p; 4: Aca-miR-212a-5p; 5: Dre-miR-205-3p; 6: Tgu-miR-155-5p; 7: oan-miR-147. Due to the different relative expression levels, the marker miRNAs were divided into two graphs with higher average ΔΔCT value (**A**) and lower average ΔΔCT value (**B**), respectively.

**Figure 7:**
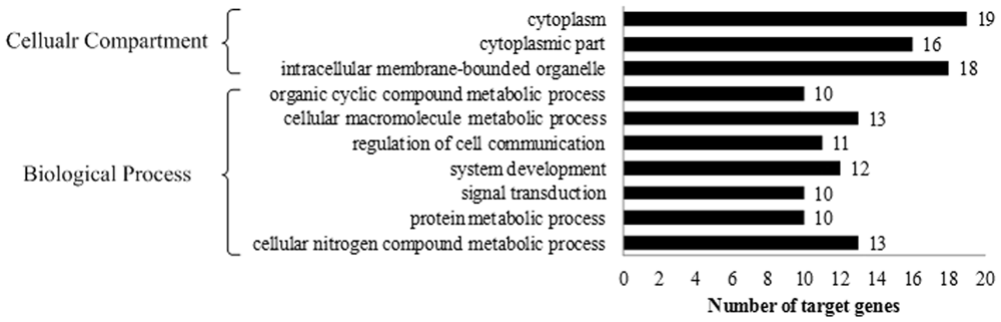
GO analysis of targets genes related to marker miRNAs.

MiRNAs has been proved to be effective signatures in many diseases and biological processes^30^. With the identification of the large amount of differentially expressed miRNAs in males and females, we tried to confirm some especial miRNAs among them as the markers for sex differentiation of *C. semilaevis*. The markers were selected from signature miRNAs because that these miRNAs showed outstanding expression differences between male and females. It is preferred that all the markers are abundant in the same sexuality for the convenience of detection. Finally, 7 miRNAs among the signature miRNAs which are abundant in males but have none or relative little expression in females were selected as the markers. Real Time quantitative PCR (RT-qPCR) was used to quantitatively measure the expression of marker miRNAs in 10 male and 10 females, respectively (Supplementary File 5). Aca-mir-100 was used as the internal reference to normalize the miRNA RT-qPCR data because that it shows similar stable high expression in both males and females. It is the first time to use aca-mir-100 as the normalization control rather than those previous used for the miRNA analysis by RT-qPCR in mammalian, such as mir-16, which has quite low expression in *C. semilaevis* exosomes. The results of RT-qPCR showed that the expression of all the marker miRNAs in ten groups of samples were significantly higher in males than females (Fig. 6), which is consistent with the results obtained by the miRNA profile analysis. The high and obvious differential expression of these miRNAs fulfilled the requirements for a good marker from the sex differentiation of *C. semilaevis*. Totally 27 genes were targeted by the 7 marker miRNAs. The target genes of dre-miR-205-3p, ssa-miR-212a-5p, bta-miR-132 and oan-miR-147 function in system development, signal transduction and sex differentiation, which proved the important role of these markers in the sexually dimorphic growth of *C. semilaevis*. GO analysis of all the targets genes for marker miRNAs were shown in Fig. 7.

## Discussion

Separation of male and female fishes is an important and difficult problem in the aquaculture of *C. semilaevis*. Many studies have focused on the influences of genes on the sex differentiation. In recent years, a few of genes have been identified associated with the development of male and female fishes, such as *Figla_tv1*, *Figla_tv2* ^45^, *GATA4* ^46^ and *CSDAZL* ^47^. However, the growth and development were not determined on any single gene but influenced by a set of genes. Lots of genes might be found to be associated with the sex differentiation but none of a single one can be the decisive element. Therefore, ignoring the united function of different genes, the molecular mechanism of the growth differences between males and females  is still ambiguous.

The methods for screening of functional genes were studied by many researchers. Wang *et al*. predicted the human infertility-related genes with the shortest path method based on the human Protein-protein Interaction Network (PIN). The candidate genes were subsequently screened through permutation test, maximum interaction scores and maximum alignment score^43^. Finally 23 putative infertility-related genes were selected among the original 373 candidate genes. Their work provides a novel direction for investigating human infertility based on the PIN and bioinformatical analysis. For *C.semilaevis*, although no PIN is available, we also found something valuable for the bioinformatical analysis on sex differentiation – miRNA expression in exosomes. Exosomes widely distribute in many kinds of physiological fluids and various organisms such as human, rat and salmon. Due to its special feature in composition, exosomes are easily to be identified and extracted. The large amount of miRNAs included in exosomes are important signatures for some diseases and special cells lines. Based on these features, the attentions focused on the study of exosomes and signature miRNAs are keeping increasing. In the study of exosomes, we found that a remarkable feature of the exosomes in *C.semilaevis* is that the miRNA dominant the nuclear acid components in exosomes. We also found that the class and amount of miRNAs were quite different in male and female *C. semilaevis*. The differentially expressed miRNAs takes about 34% (246/723) and 37% (247/662) of male and female total miRNAs. This remarkable difference enables miRNA an effective index for distinguishing the male and female fishes. Therefore, the investigation of miRNA may supply an effective way for the study of the decisive gene set for the sex differentiation due to its function on regulation of multiple genes.

In this study, we found 270 differentially expressed miRNAs and identified 20 signature miRNAs. Subsequently 7 miRNAs which were abundant in males but have none or relative little expression in females were selected from signature miRNAs as the markers for sex identification. Totally 27 genes were found to be the targets of these markers, which comprised a potential gene set for sex differentiation. Several of these genes have been proved to play important roles in the growth or development process in other organisms. *npy2r*, a target gene of ssa-miR-212a-5p, is found working in close coordination with the melanocortin and corticotropin-releasing hormone systems to modulate body weight of rats^48^. The activity of *eno2*, a target gene of bta-miR-132, is found essential for the growth and development of plants^49^. In addition, a hemogen gene *hemgn* is identified as another target gene of bta-miR-132, with its homolog proved to be specifically involved in testis differentiation in early chicken embryos and thus involved in chicken sex determination^50^. Furthermore, according to GO term categories generated from all the targeted genes for the marker miRNAs, 17 (about 63%) target genes function in system development, signal transduction or regulation of cell communication, which proved on the other hand the important role of these target genes in the regulation of the growth and development in male and female fishes. These results in some extent certified the reliability of the predicted target genes. Of course, more experiments are still needed for the further confirmation of the function of these genes in *C. semilaevis*.

## Conclusion

In this work, we proved the existence of exosomes in *C. semilaevis* serum, which is the first discovery of exosomes in fish serum. MiRNAs were found to dominant in the total RNA compositions in exosomes and signature miRNAs from exsomes were identified for distinguishing male and female *C. semilaevis* by miRNAs profile sequencing and analysis. The target gene prediction and annotation indicate that the signature miRNAs may influence the sexuality of *C. semilaevis* indirectly through the regulation and metabolism of macromolecular, nitrogen and heterocycle, as well as acting on the sex differentiation process directly. For the convenience of sex detection, 7 markers with 27 target genes were finally selected from signature miRNAs and verified by RT-qPCR, which offered a novel method for the study of sex differentiation of *C. semilaevis* in the reproduction and cultivation.

## Methods

### Isolation of exosomes in *C. semilaevis*

**Figure 8:**
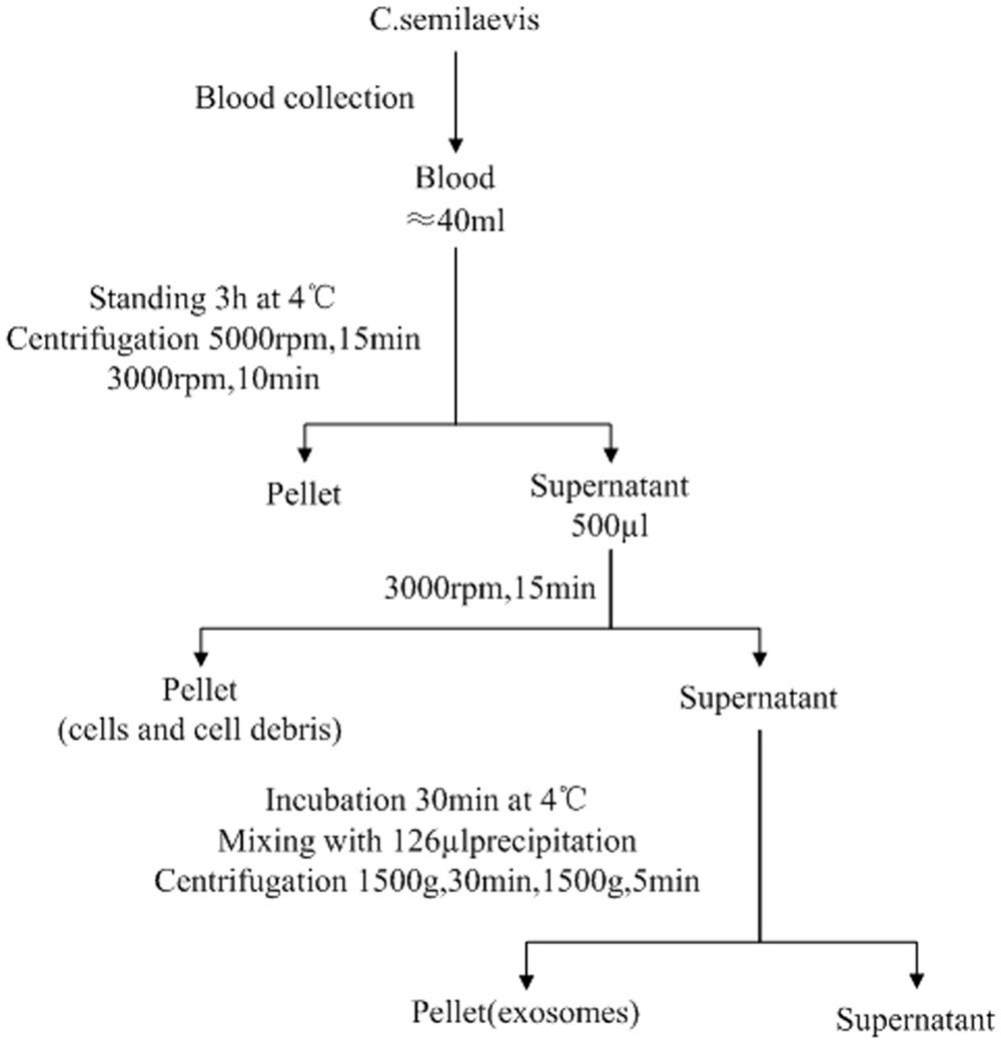
Flowchart for exosomes isolation from *C. semilaevis* serum.

*C. semilaevis* specimens were obtained from Haifa Ltd., Tianjin, China. Ten male and ten female 3-year-old fishes were selected. The body length and weight were shown in Table 1. The lengths of females were averagely 202 mm longer than males, and the weights of females were averagely 733 g heavier than males, which reflect the obvious sexually dimorphic growth with the females growing much faster than males. The fish were kept at 25 °C in aerated seawater for a week before using. Blood drawn from 10 tails of male and female fishes respectively was collected. For exosome isolation, the blood from ten male fishes was merged, and so did the female fishes. Exosomes in blood were isolated with differential centrifugation (Fig. 8). The isolated exosome pellet was resuspended in 1/10 of original volume using sterile water prepared for the following detection and analysis.

**Table 1:** The length and weight of female and female fishes.

Female		Male	
length (mm)	weight (g)	length (mm)	weight (g)
520	960	315	190
535	1000	345	210
515	1020	310	160
525	800	313	190
510	820	313	210
520	890	320	210
550	1000	340	210
515	1030	320	210
515	1050	325	190
500	720	323	180

### Electron microscopy

Approximately 50 μl of prepared exosomes was loaded onto formvar carbon-coated 300 mesh copper grids and adsorb for 10 min. The adsorbed exosomes were then negatively stained with 3% phosphotungstic acid and dried at room temperature for 20 min. Subsequently, the exosomes were observed under a transmission electron microscope (Olympus Software Imaging Solutions) at 120.0 kV and the images of exosomes were captured by a digital camera.

### Nanopartical Tracking Analysis

The purified exosome pellet was subjected to size and concentration measurement by NanoSight LM10 (Malvern Instruments, Westborough, MA) at the OSU Comprehensive Cancer Center (OSUCCC) Analytical Cytometry Shared Resource. NanoSight with nanoparticle tracking analysis (NTA) software visualizes and analyzes nanoparticles in liquids from 10 to 2000 nm by relating the rate of Brownianmotion to particle size^51^. Vesicles were visualized by light scattering using a light microscope. NTA software tracks the motion of individual vesicles and calculates their sizes and total concentration with a video taken. The following software settings were employed and constant for all samples: detection threshold, 10 Multi; blur, auto; minimum track length, auto; and minimum expected particle size, 10 nm. The measurement of exosome concentration performed in this study calculated particle size on a particle-by-particle basis in a 60-second video recorded at a frame rate of 25 frames/s to provide accuracy and statistics for the analysis. The video was subsequently analyzed with NTA 2.3 software to obtain the size information of the exosomes, including the mean, mode and concentration.

### Western Blotting Analysis

The exosomal proteins were separated via sodium dodecyl sulfate-polyacrylamide gel electrophoresis (SDS-PAGE) and transferred to PVDF membranes (Millipore Corp. Bedford, MA). The membranes were blocked in Tris-buffered saline (TBS) containing 5% nonfat milk at 28 °C for 2 h and then incubated with specific primary antibodies at 4 °C overnight (1:1000, System Biosciences, USA). The membranes were washed 2 times with TBST (TBS containing 0.1% Tween-20) and then incubated with a peroxidase-labeled anti-rabbit secondary antibody (1:1000, System Biosciences, USA). After washed 4 times with TBST, the membranes were visualized by chemiluminescence using the enhanced chemiluminescence (ECL) western blot analysis system (Novex ECL Chemiluminescent Substrate, Life Technologies). Brightness and contrast of images were adjusted by the software of Adobe Photoshop (Adobe Systems).

### RNA library construction and sequencing

The total RNAs were extracted and sequenced by RIBOBIO Ltd. The RNA samples from male and female *C. semilaevis* were sequenced separately. The miRNA profiles were sequenced by Illumina HiSeq^TM^ 2500. The clean RNA reads were annotated by aligning to the known ncRNAs with Burrows-Wheeler Aligner (BWA) software^52^. The un-mapped RNA reads were used for the prediction of novel miRNAs with Mireap software.

The miRNA profiles for male (TJFISH-1) and f**e**male *C. semilaevis* (TJFISH-2) were sequenced and compared. The miRNA clean reads were annotated to the known miRNAs in miRBase database and the miRNAs with less than 10 total counts were ignored. Significant miRNA changes were selected based on the following criteria: (i) statistical significance—miRNA expression changes were identified using a P-value threshold of 0.01; and (ii) fold change expression—a minimum 2-fold difference in either direction was required.

### Bioinformatics analysis on target matching

The target genes of selected miRNAs were predicted with three software: miRanda^53^, PITA^54^ and RNAhybrid^55^. Genes with Total Score ≥ 90 and Total Energy < −15 kmol were predicted as targets with miRnada. Genes with combined interaction energy < 10 were considered as targets in PITA. Genes with minimum free energy ≤ −20 were recognized as targets in RNAgybrid. Only targets that were found with all three software were identified to be the target genes of miRNAs. GO annotation of target genes were obtained from Gene Ontology database with p-value threshold of 0.05.

### Real time quantitative PCR

Exosomal miRNA expression was assayed using real time Quantitative polymerase chain reaction (RT–qPCR) in serum sample of Female and male fishes. Total RNAs (10 ng) from 2 samples were used to reverse transcription. The reactions from total RNA using Taqman MicroRNA primers specific for PCRs were performed using Thermal Cycler under the following conditions: 30 min at 16 °C, 30 min at 42 °C, and 5 min at 85 °C. The products stored at −20 °C for later use or immediately processed according to the manufacturer’s protocol.

Quantitative PCR was performed in 96-well reaction plates with an ABI 7500 Fast Real-Time PCR System (Applied Biosystems, Foster City, USA). No template controls were used to evaluate background signal. The RT-qPCR program consisted of 50 °C for 2 min, 95 °C for 10 min, followed by 40 cycles each of denaturation at 95 °C for 15 s and annealing and extension for 60 s at 60 °C. The expression level was used as a stable endogenous control for normalization. Each sample was run in three duplicates and relative quantification of miRNA expression was calculated using the 2^−ΔΔCt^ method. Aca-mir-100, which has similar expression in males and females, was used as the internal reference.

### Ethics Statement

Haifa Ltd.(Tianjin, China) provided consent for use of live *C. semilaevis* for research. The methods for *C. semilaevis* research were carried out in accordance with the relevant guidelines and regulations. The protocols were approved by the College of Life Sciences, Zhejiang University and Tianjin Key Laboratory of Animal and Plant Resistance/College of Life Sciences, Tianjin Normal University.

## Electronic supplementary material

Dataset 1

Dataset 2

Dataset 3

Dataset 4

Dataset 5
